# Faces in the dark: interactive effects of darkness and anxiety on the memory for threatening faces

**DOI:** 10.3389/fpsyg.2014.01091

**Published:** 2014-10-02

**Authors:** Satoshi F. Nakashima, Yuko Morimoto, Yuji Takano, Sakiko Yoshikawa, Kurt Hugenberg

**Affiliations:** ^1^Human Information Science Laboratory, NTT Communication Science LaboratoriesAtsugi, Japan; ^2^CREST, Japan Science and Technology AgencyAtsugi, Japan; ^3^Department of Evolutionary Studies of Biosystems, School of Advanced Sciences, The Graduate University for Advanced StudiesHayama, Japan; ^4^Kokoro Research Center, Kyoto UniversityKyoto, Japan; ^5^Department of Psychology, Miami UniversityOxford, OH, USA

**Keywords:** darkness, anxiety, facial expression, face memory

## Abstract

In the current research, we extend past work on the effects of ambient darkness and threat to the domain of memory for expressive faces. In one study, we examined the effects of ambient darkness and individual differences in state anxiety on memory of unfamiliar expressive faces. Here, participants were seated in either a dark or light room and encoded a set of unfamiliar faces with angry, happy, and neutral facial expressions. A subsequent recognition task revealed an interactive effect of ambient darkness, anxiety, and target expression. Highly anxious participants in ambient darkness had worse memory for angry faces than did low-anxiety participants. On the other hand, the recognition performance for happy faces was affected neither by the darkness nor state anxiety. The results suggest not only that ambient darkness has its strongest effect on anxious perceivers, but also that person × situation effects should be considered in face recognition research.

## Introduction

Facial expressions are meaningful social signals for human interaction in daily life. Numerous researchers have indicated that humans infer expressers' emotional states (Ekman, 2003), behavioral tendencies (Frijda, 1995), or intentions (Fridlund, 1994) from facial expressions. Particularly, the ability to detect a threatening signal, such as anger from the face of another individual, is a crucial skill for avoiding the risk of attack from an angry person or maintaining an appropriate relationship by placating the person. Angry faces are easily detected in the environment (e.g., Hansen and Hansen, 1988), especially when looming toward the self (see Adams et al., 2006), in part because they appear to arrest attention. For example, Springer et al. (2007) demonstrated that the presentation of an angry face produces a stronger startle reflex than does a happy or neutral face (Springer et al., 2007). Perhaps not surprisingly, given the adaptive advantages to responding quickly to threats, angry expressions in the environment also serve to quickly engage avoidance behaviors as well (Marsh et al., 2005).

Recently, researchers have given considerable attention to the effects of facial expressions as emotional information on memory for facial identity (D'Argembeau et al., 2003; Johansson et al., 2004; Sergerie et al., 2005; Anderson et al., 2006; D'Argembeau and Van der Linden, 2007). The findings from previous studies, however, have been inconsistent. Some studies have shown that negative facial expression enhances memorability of facial identity more than that of positive facial expression (Johansson et al., 2004; Sergerie et al., 2005), whereas others have shown opposite results (D'Argembeau et al., 2003; D'Argembeau and Van der Linden, 2007) or null effect of facial expression (Anderson et al., 2006; Ishai and Yago, 2006).

Perhaps these inconsistencies in the effects of facial expressions on facial identity are caused in part because few studies have investigated how contextual information can modulate how faces are perceived. Indeed, the perceived meaning of a facial expression can be quite sensitive to the context in which it is encountered (see Carroll and Russell, 1996). As examples of this, a variety of recent studies have shown that various contexts, such as an emotional scene (Righart and de Gelder, 2008), odors (Leppänen and Hietanen, 2003), body posture (Meeren et al., 2005; Van den Stock et al., 2007; Aviezer et al., 2008), hand movements (Hietanen and Leppänen, 2008), and in-group vs. out-group distinction (Hugenberg, 2005) can all affect our perceptions of others' facial expressions of emotion. More important for the current work, recent research has also shown that emotional contextual information affect recognition memory for unfamiliar faces. Specifically, Van den Stock and de Gelder (2012) examined how emotional body posture and emotional background scene independently combined with faces to alter recognition performance for faces. The results provided the evidence that both emotional body and background scenes hamper recognition performance for facial identities. Taken together, the extant research clearly indicates that not only can contextual information influence how faces are perceived and remembered.

In the current research, we extend this perspective that face perception and memory is sensitive to contextual influences to an investigation of ambient darkness, testing the hypothesis that ambient darkness—itself a potential cue for danger—may interact with individual differences in anxiety to influence memory for faces with angry expressions. To this end, we first discuss recent findings indicating that ambient darkness can serve as a danger cue, and that ambient darkness can interact with individual differences in susceptibility to threat (e.g., anxiety) to influence social perception. We then present one study in which we investigate the interactive effects of ambient darkness and state anxiety on memory for angry, happy, and neutral expressive faces.

### Ambient darkness, anxiety, and threatening faces

Cross culturally, environmental or ambient darkness is associated with danger, evil, and threat (see Schaller et al., 2003). Indeed, across a variety of experimental studies, ambient darkness appears to be a signal for threat. For example, darkness increases the magnitude of the startle reflex (eye blink) to auditory stimuli (Grillon et al., 1997, 1999). Relatedly, ambient darkness also increases the likelihood that perceivers will activate danger-related stereotypes. For example, Schaller et al. (2003) demonstrated that ambient darkness triggers the activation of anti-Black stereotypes, a strongly danger-related stereotype, especially for perceivers who believe the world is dangerous. Thus, participants who had a greater individual difference susceptibility to believing the world is threatening (e.g., agreeing with statements such as “Every day, as our society becomes more lawless and bestial, a person's chances of being robbed, assaulted, and even murdered go up and up”), showed stronger activation of danger-related stereotypes when in ambient darkness. Not only does this indicate that darkness can serve as a threat cue in social contexts, but it also indicates that ambient darkness can interact with individual differences in susceptibility to threat to generate person × situation effects in processing social information.

### The present study

In the current research, we sought to investigate the influence of ambient darkness and individual differences in anxiety on memory for expressive faces. Although past research has demonstrated that ambient darkness can influence person perception, how might darkness influence memory for expressive faces? To our knowledge, no past research has addressed this question directly, however, past research can allow us to draw clear hypotheses. First, a great deal of research has shown that individual differences in anxiety are closely related to the processing of threatening faces (Bradley et al., 1998, 1999; Fox et al., 2001). For instance, highly anxious people at first more quickly direct their attention to threatening faces than low-anxiety people (e.g., Bradley et al., 1998) but later tend to avoid such faces (e.g., Rohner, 2002). Although this vigilance-avoidance effect typically found in attention can lead perceivers to accurately detect the environmental threat, it can actually degrade memory for that stimulus (e.g., Ackerman et al., 2009). Based on this logic, we predict that anxious perceivers will demonstrate worse memory for angry expressions. However, we believe that this will be qualified by ambient darkness. Given that ambient darkness can signal danger, we predict this will potentiate the tendency for anxious perceivers to have difficulty remembering angry faces.

Thus, although past research implies a close relationship among ambient darkness, anxiety, and memory for angry faces, to our knowledge, no study has yet examined these interactions directly. In the present study, we examined whether ambient darkness and anxiety affect memory for angry, but not happy or neutral faces. To investigate this, participants were seated in a dark or a well-lit room, and were first asked to rate the expressivity of faces displaying angry, happy, and neutral facial expressions. Three minutes later, all participants were again shown target faces interspersed with previously unseen distractor faces, and were asked to indicate whether they had viewed each face before or not.

Although not our central focus, we also examined the effect of participant's sex on each emotional faces as control variable, because several previous studies have shown the presence of sex difference on our target variables such as anxiety (Bekker and van Mens-Verhulst, 2007), face memory (Rehnman and Herlitz, 2007) and recognition of facial expression (Thayer and Johnsen, 2000). In particular, one study showed that recognition performance for unfamiliar faces in female participants was superior to those in male participants (Rehnman and Herlitz, 2007); therefore, we tested for the possibility that female participants may demonstrate superior face memory than male participants.

## Methods

### Participants

Forty-four undergraduates from Kyoto University participated in the experiment (20 men and 24 women; mean age of 19.57). Of these, 21 (11 men, 10 women) were assigned to the darkness condition, and 23 (9 men, 14 women) were assigned to the light condition. The data of 1 female participant in darkness condition was removed because of too many missing values. This study was approved by the faculty in the Department of Cognitive Psychology in Education at Kyoto University, and was conducted in accordance with the ethical code of the Japanese Psychological Society.

### Materials and apparatus

From the ATR Japanese Face Database (Ogawa et al., 1997), we selected 144 face photographs of 48 Japanese individuals (24 females and 24 males) who each expressed a happy, angry, or neutral facial expression. None of the faces had any distinctive marks or wore glasses or a beard. We performed grayscale transformation on all of the photos. The head size of each individual was equalized, and the background of each photo was cropped using Adobe Photoshop software. These photos were validated in a previous study (see Nakashima et al., 2012).

The images of the 48 different individuals were divided into two sets of 24 (12 males and 12 females). The faces from one set were studied in the learning phase of the experiment; the faces from the second set were used as unstudied items and appeared as distracters in the test phase of the experiment. Of 24 individuals in each set, we allotted 8 individuals (4 males and 4 females) to each facial expression (angry, happy and neutral). The allocation of the two sets to learning or test phase and the allocation of individual to each facial expression were counterbalanced across participants.

All of the facial photographs were projected on a PC monitor by SuperLab 4.0 (Cedrus).

### Questionnaires

In the study, participants were asked to rate the Japanese version of the State Anxiety Inventory (STAI) (Shimizu and Imae, 1981), which was translated from Spielberger et al.'s (1970) State-Trait Anxiety Inventory. This questionnaire is a frequently used and well-validated anxiety scale (α = 0.87).

### Procedure

Participants were tested individually in a light shielded, acoustically shielded room. In the light condition, the experimenter turned on the room lights and closed the door before giving the instructions for the experiment. In the dark condition, the experimenter turned off the room lights and closed the door before giving the instructions. The participants were told that the reason the light had been switched off was so that they could focus their attention only on the monitor without any distractions.

The experiment comprised a learning phase, a retention interval, and a surprise test phase. In the instructions, participants were informed about the task of rating on facial photographs in learning phase, but not that a memory test would follow the learning phase (incidental learning). After instructions had been given, participants practiced rating facial expressions with facial photographs that were not used in the primary trials. A practice session comprised ten trials. After the practice, the experimenter exited the experimental room, and participants started the experiment at their own pace.

During the learning phase, a fixation cross was displayed for 200 ms. Each face was then shown to the participants for 3000 ms, after which the fixation cross vanished. Scrambled masks were then presented for 60 ms, followed by the presentation of a 9-point Likert scale for the facial expression rating (1 for happy to 9 for angry). Participants were asked to rate the “happiness-angriness” of the face by pressing one of the numeric keys on the keyboard. They were asked to perform this task at their own pace and that the next trial would be initiated by pressing any key on the keyboard. A set of 24 photographs of individual that include eight individual for each expression were presented as target stimuli.

Immediately after the learning phase, participants were asked to rate the STAI-state on the monitor of the PC. Each item and a four-point scale were presented on the center of the monitor. Participants were asked to respond by using numeric keys 1 through 4 on the keyboard. This task was used for two reasons: to measure the participant's state anxiety in each condition and to prevent participants from rehearsing the faces during the retention interval.

Following the 3-min retention interval, participants completed the recognition test. They were informed that they would be shown a series of faces, some of which would be those of the individuals whose faces they had seen in the earlier phase of the experiment. Each face was presented on the computer screen and participants were asked to respond “yes” if they had seen the face before; otherwise they were asked to respond “no.” Responses were made by pressing the F key for a “yes” response and the J key for a “no” response on a standard keyboard. Faces were displayed on the screen until participants made their response. Twenty-four target faces and 24 distracter faces (8 individuals for each expression) were presented.

## Results

### Emotion ratings

**Figure 1:**
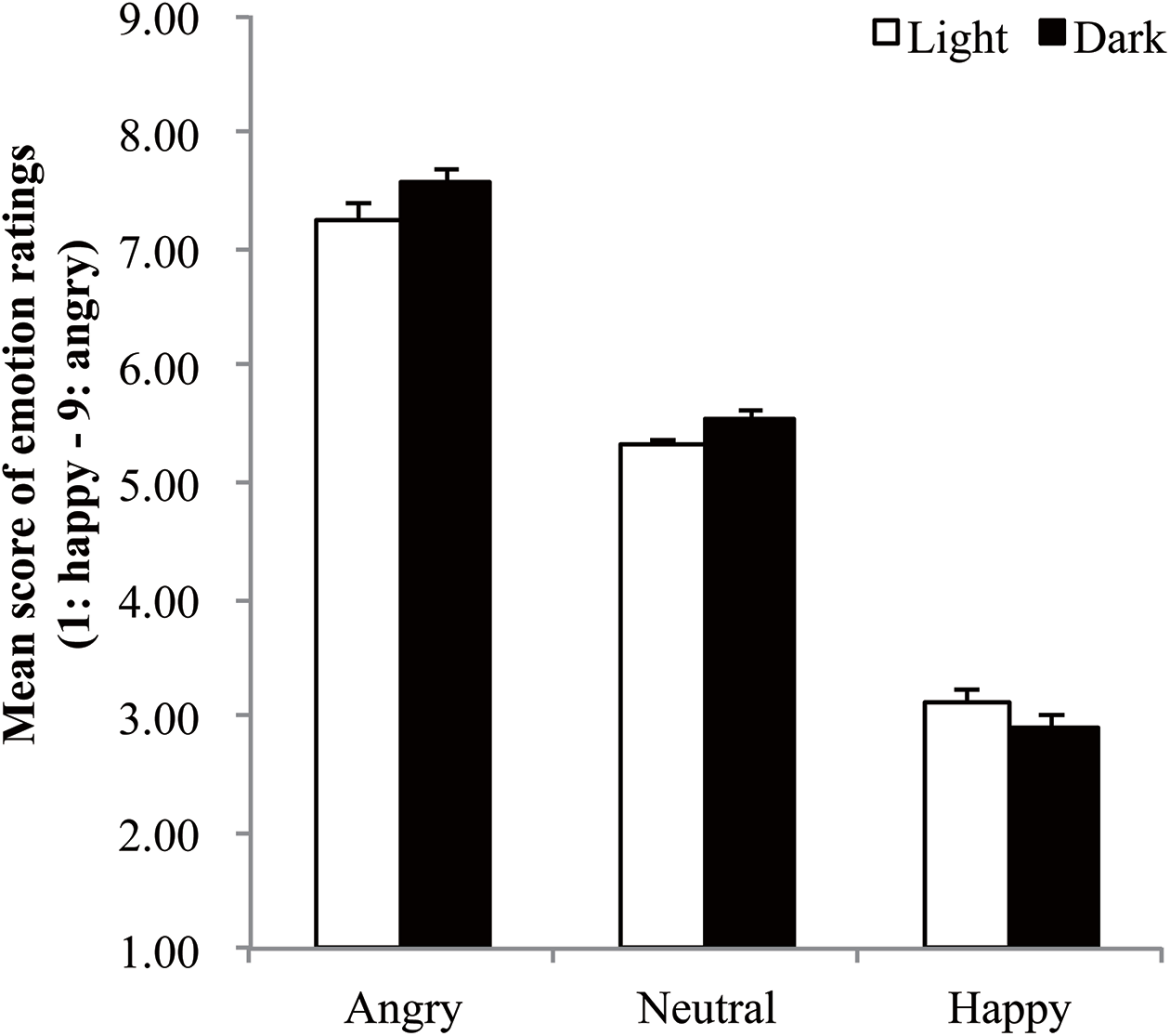
**Mean score of emotion ratings for each emotional expression as a function of the darkness**. Black bars represent ratings for dark condition; white bars represent ratings for light condition. Error bars indicate standard error.

To ensure that the angry, happy, and neutral faces were indeed perceived as differentially expressive, we first computed average emotion ratings of each emotional face for each participant (higher values indicate higher angry ratings). The mean score of emotion ratings for each condition are shown in Figure 1. These averages were then submitted to a 2 (condition: light, dark) × 3 (facial expression: angry, happy, neutral) mixed ANOVA. This ANOVA yielded the predicted main effect of facial expression, *F*_(2, 82)_ = 801.97, *p* < 0.001, η^2^ = 0.93. Participants perceived angry faces as angrier than neutral faces and happy faces (angry: *M* = 7.41, *SE* = 0.09; neutral: *M* = 5.43, *SE* = 0.05; happy: *M* = 3.01, *SE* = 0.07). The differences between each pair of facial expression were all significant (all *p*s < 0.001). The result indicates that participants perceived each picture of facial expression in expected emotion categories.

The main effect of lighting condition was marginally significant, *F*_(1, 41)_ = 3.29, *p* = 0.077, η^2^ = 0.001. Participants in dark condition perceived facial images as angrier than those in light condition (dark: *M* = 5.34, *SE* = 0.05; light: *M* = 5.22, *SE* = 0.05). In addition, the interaction between condition and facial expression was also marginally significant, *F*_(2, 82)_ = 3.04, *p* = 0.053, η^2^ = 0.003. Participants in dark condition perceived angry faces as more expressive than those in light condition (dark: *M* = 7.56, *SE* = 0.11; light: *M* = 7.26, *SE* = 0.13). In contrast, the differences between light and dark condition for happy and neutral faces did not reach statistical significance (*p*s > 0.05). In line with recent findings indicating both that context influences expression perception, and that darkness serves as a threatening context, these results indicate that the perception of angry faces was somewhat altered by darkness. We, therefore, decided to conduct analyses on recognition memory performance with including the data of emotion rating as a control variable.

### Recognition performance

We used a signal detection measure of discriminability *d*′ to determine performance in the facial recognition memory task. One advantage of this statistic is that the value is independent of an observer's threshold for making a response (Wickens, 2002). Table 1 shows the means, standard deviations (SD), and correlations among variables.

**Table 1:** **Means and Standard deviations (SD) of each variable and Correlations between each variable (*N* = 43)**.

		***M***	***SD***	**Correlations**		
				**1**	**2**	**3**
1	Sex of participants	–	–			
2	Condition	–	–	−0.16		
3	State anxiety	2.11	0.42	−0.08	−0.07	
4	Angry (d′)	1.68	0.74	0.20	−0.24	−0.34^*^
5	Happy (*d*′)	1.44	0.73	0.14	−0.16	−0.17
6	Neutral (d′)	1.49	0.59	0.39^*^	−0.22	−0.36^*^

*Note: The values under rows of 1–3 represent correlation coefficients between each variable. Correlation between recognition performances for each facial expression was irrelevant to following analysis, so that we did not show them in the table*.

* *p < 0.05*.

Preliminary correlation analysis showed that recognition performances (*d*′) of angry and neutral faces were negatively correlated with state-anxiety (angry: *r* = −0.34, *p* = 0.03; neutral: *r* = −0.36, *p* = 0.02). In contrast, there was no significant correlation between recognition performance (*d*′) of happy faces and state anxiety (*r*= −0.17, *p* = 0.27). We did not find significant relationships between manipulated context and recognition performance of each facial expression.

**Figure 2:**
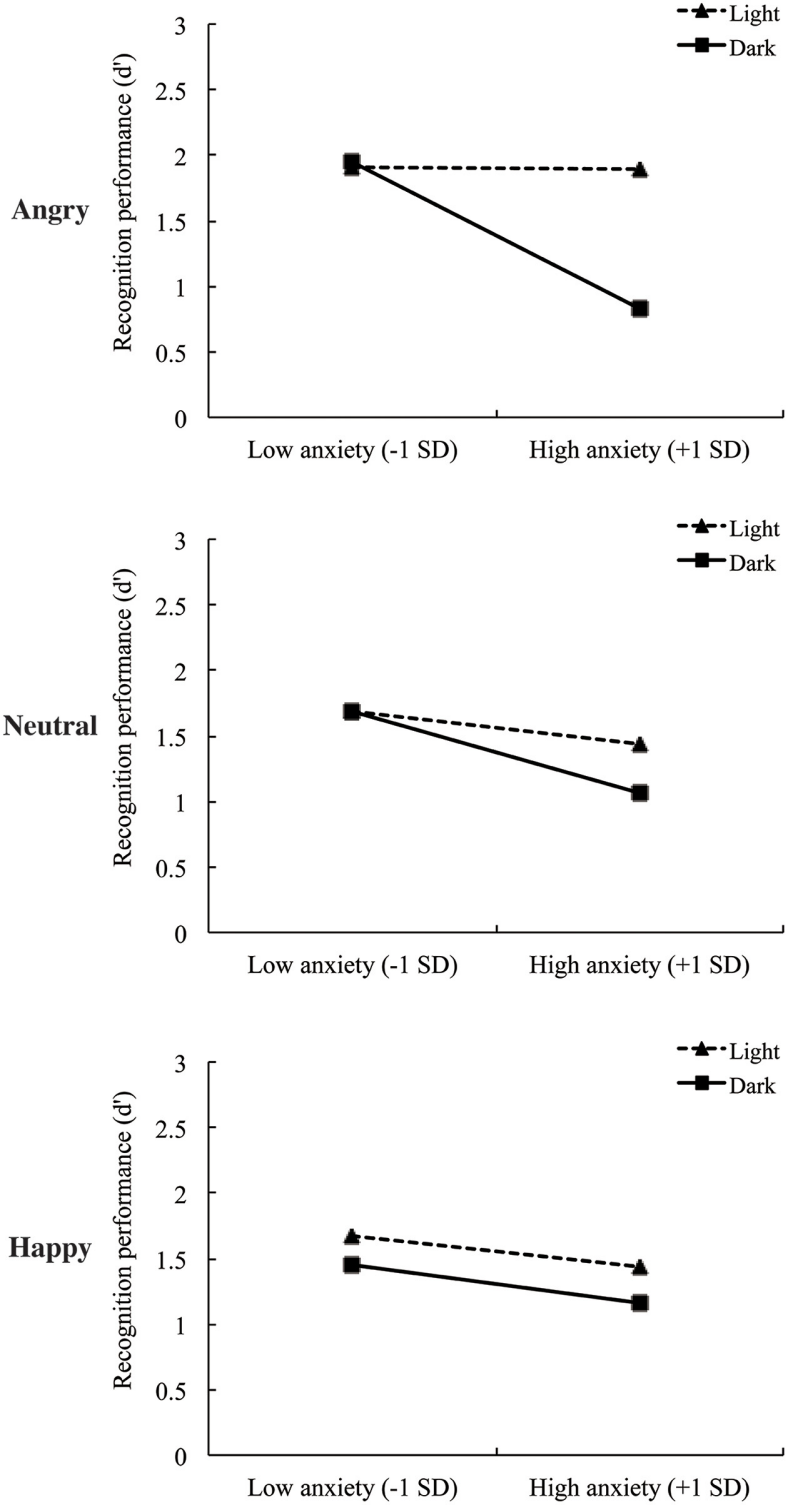
**Regression lines of recognition performance (d′) for each facial expression as a function of the darkness and state anxiety**. The slopes illustrated in this graph were calculated with assigning the value of ±1 *SD* of state anxiety to regression equation in accordance with Cohen and Cohen (1983).

Statistical analyses on recognition performance (*d*′) were conducted by hierarchical regression. To test our hypothesis, we regressed recognition performance on the sex of participants and emotion ratings for each face during encoding phase as control variable, the manipulated context values, state anxiety score, and their interaction term. For analyses, values of 1 and 0 were assigned to the two levels of the manipulated context (dark = 1; light = 0) and the sex of participants (female = 1; male = 0). To reduce the effects of multicollinearity, the state anxiety score was standardized (Aiken and West, 1991). All four predictors were entered hierarchically in three sets of regression analyses. Recognition performance was analyzed separately with respect to each facial expression condition. Results of these three analyses are presented in Table 2, Figure 2 and Figure S1.

**Table 2:** **Results of hierarchical regression analyses predicting recognition performance (d′)**.

		**Model 1: base model**		**Model 2: main effects model**		**Model 3: full model with interaction**	
Angry face	Step 1: Control variables						
	Sex of participants	0.26	(0.23)	0.10	(0.22)	−0.05	(0.10)
	Emotion rating	0.13	(0.20)	0.27	(0.19)	0.30	(0.18)
	Step2: Main effects						
	Darkness			−0.47^*^	(0.22)	−0.51^*^	(0.10)
	State anxiety			−0.62^*^	(0.25)	−0.02	(0.23)
	Step3: Interaction						
	Darkness × state anxiety					−1.30^*^	(0.24)
	Overall model R^2^	0.05		0.24		0.37	
	Adjusted R^2^	0.00		0.16		0.28	
	ΔR^2^			0.19		0.13	
	ΔF			4.81^*^		7.38^*^	
	Overall F	1.05	(*df* = 2, 40)	3.03^*^	(*df* = 4, 38)	4.31^**^	(*df* = 5, 37)
Neutral face	Step 1: Control variables						
	Sex of participants	0.46^**^	(0.17)	0.39^*^	(0.16)	0.35^*^	(0.17)
	Emotion rating	−0.11	(0.27)	−0.17	(0.28)	−0.20	(0.28)
	Step2: Main effects						
	Darkness			−0.19	(0.17)	−0.19	(0.17)
	State anxiety			−0.50^*^	(0.20)	−0.31	(0.26)
	Step3: Interaction						
	Darkness × state anxiety					−0.43	(0.39)
	Overall model R^2^	0.16		0.30		0.32	
	Adjusted R^2^	0.11		0.23		0.23	
	ΔR^2^			0.15		0.02	
	ΔF			3.96^*^		1.20	
	Overall F	3.69^*^	(*df* = 2, 40)	4.10^**^	(*df* = 4, 38)	3.54^*^	(*df* = 5, 37)
Happy face	Step 1: Control variables						
	Sex of participants	0.20	(0.23)	0.13	(0.23)	0.12	(0.24)
	Emotion rating	−0.06	(0.24)	−0.13	(0.24)	−0.13	(0.25)
	Step2: Main effects						
	Darkness			−0.25	(0.24)	−0.25	(0.24)
	State anxiety			−0.32	(0.27)	−0.29	(0.38)
	Step3: Interaction						
	Darkness × state anxiety					−0.05	(0.57)
	Overall model R^2^	0.02		0.08		0.08	
	Adjusted R^2^	−0.03		−0.02		−0.05	
	ΔR^2^			0.05		0.00	
	ΔF			1.11		0.01	
	Overall F	0.42	(*df* = 2, 40)	0.77	(*df* = 4, 38)	0.60	(*df* = 5, 37)

*Note: The table represents unstandardized regression coefficients (standard errors in parentheses)*.

* *p < 0.05*,

** *p < 0.01*.

### Angry face

In Model 1, the base model, we regressed recognition performance on the sex of participants and emotion rating as our control variables. The overall results indicated that the control variables explained only 5% of the variance in recognition performance. In Model 2, we entered the darkness and state anxiety as independent variables in regression analysis. The results yielded a significant effect of state anxiety: state anxiety was negatively related to recognition performance [*b* = −0.62, *SE* = 0.25, *t*_(38)_ = −2.49, *p* = 0.02]. In addition, the effect of darkness reached the statistical significance [*b* = −0.47, *SE* = 0.22, *t*_(38)_ = −2.09, *p* = 0.04]. This step explained approximately 19% of the incremental variance in recognition performance (Δ*R*^2^ = 0.19, *p* = 0.014). Overall, Model 2 yielded an *R*^2^ of 0.24.

In Model 3, we tested the prediction that state anxiety moderates the relationship between darkness and recognition performance. The results showed that the interaction between darkness and state anxiety was significant [*b* = −1.30, *SE* = 0.48, *t*_(37)_ = −2.72, *p* = 0.01]. This step explained 13% of the incremental variance in recognition performance (Δ*R*^2^ = 0.13, *p* = 0.01). Overall, Model 3 yielded an *R*^2^ of 0.37. Simple slope analyses (Aiken and West, 1991) revealed that recognition performance was negatively related to darkness in high state anxiety [*b* = −1.05, *SE* = 0.30, *t*_(37)_ = −3.53, *p* = 0.001], whereas recognition performance was unrelated to darkness in low state anxiety [*b* = 0.04, *SE* = 0.28, *t*_(37)_ = 0.14, *p* = 0.89]. Moreover, recognition performance was negatively related to state anxiety in the dark condition [*b* = −1.32, *SE* = 0.35, *t*_(37)_ = −3.82, *p* < 0.001], whereas recognition performance was unrelated to state anxiety in the light condition [*b* = −0.02, *SE* = 0.32, *t*_(20)_ = −0.07, *p* = 0.94]. These simple effects are depicted in Figure 2A.

### Neutral face

We conducted hierarchical regression analyses of recognition performance for neutral faces, as in the case for angry faces. In Model 1, the results yielded a significant effect of the sex of the participants: female participants showed better recognition performance [*b* = 0.46, *SE* = 0.17, *t*_(41)_ = 2.70, *p* = 0.01]. The overall results indicated that control variable explained approximately 16% of the variance in recognition performance. In Model 2, the results yielded a significant effect of state anxiety: state-anxiety negatively correlated with recognition performance [*b* = −0.50, *SE* = 0.20, *t*_(39)_ = −2.59, *p* = 0.014], whereas the effect of darkness was not significant [*b* = −0.19, *SE* = 0.17, *t*_(39)_ = −1.07, *p* = 0.29]. This step explained approximately 15% of the incremental variance in recognition performance (Δ*R*^2^ = 0.15, *p* = 0.03). Overall, Model 2 yielded an *R*^2^ of 0.30.

In Model 3, we tested the prediction that state anxiety moderates the relationship between darkness and recognition performance and found that the interaction of darkness and state anxiety was not significant [*b* = −0.43, *SE* = 0.39, *t*_(38)_ = −1.10, *p* = 0.28] (Figure 2B). This step explained only 2% of the incremental variance in recognition performance (Δ*R*^2^ = 0.02, *p* = 0.28).

### Happy face

Finally, the hierarchical regression analyses of recognition performance for happy faces revealed neither significant main effects nor interactions (all *p*s >0.10) (Figure 2C). This suggests that the interactive effects of context and state anxiety were specific to angry faces rather than general effects for emotional stimuli.

## Discussion

The purpose of this study was to investigate whether ambient darkness and anxiety affect the recognition performance for unfamiliar faces, especially for angry faces. To our knowledge, this experiment provides the first evidence of an interactive effect of darkness and anxiety on face memory. In particular, the more anxious participants felt, the less they were able to memorize angry faces correctly in the dark, a relationship not observed in the light.

More critically, we found that darkness modulated the impact of anxiety for angry faces but not for happy and neutral ones. This result means that interaction between darkness and anxiety are not effective on face memory generally but does affect memory for angry faces specifically. This finding is consistent with previous studies showing that darkness can signal danger, and that darkness can interact with individual differences in susceptibility to danger signals in the processing of social information (e.g., Schaller et al., 2003). In addition, the results indicate that the poor recognition performance in the dark room is not simply attributable to low visibility during face encoding. If low visibility had caused the poor recognition performance, a similar effect would have been found for all of the faces.

One possible reason the recognition performance for angry face was impaired in participants who felt high anxious in the dark room is inhibition of attention to angry faces during face encoding. In fact, the results of the data for emotion ratings showed that participants in dark condition perceived angry faces as angrier than those in light condition. Highly anxious participants in the dark room, therefore, could not pay attention to angry faces accurately in comparison with low anxiety participants. In this connection, Horley et al. (2003) showed that highly anxious social phobics could not pay attention to angry faces, especially around the eyes of them (Horley et al., 2003). This result implies that social phobics might not encode angry faces into long-term memory appropriately, because the area around the eyes is a crucial part for face memory (McKelvie, 1976). Moreover, a number of studies have indicated that high stress impairs memory of the details of an event (Christianson, 1992). Takahashi et al. (2004) have shown that social stress decreases performance for memory of face-name association. Taken together, it is considered that angry faces that appeared in the dark might put participants in a strong fearful state. This might distract participants from angry faces and thereby impair the performance for angry faces. We should note, however, that the regression analysis showed that the score of emotion rating did not affect the recognition performance of angry faces, so that we could not verify the possibility directly. Further studies are needed in order to confirm the possibility.

We found that state anxiety, independently of darkness, negatively predicted the recognition performance for neutral faces as it is for angry faces: Participants who felt highly anxious could not recognize neutral faces, compared to those who felt low levels of anxiety. This result itself is not surprising, because previous studies have already shown that induced high anxiety decreases the ability to identify neutral faces (e.g., Attwood et al., 2013). On the other hand, anxiety did not predict the performance for happy faces. This means that anxiety does not necessarily reduce recognition performance for all kinds of faces and, in particular, it might be irrelevant to the processing of positive emotional faces.

Moreover, we found that the sex of the participants predicted recognition performance for neutral faces: Female participants, relative to male participants, showed better recognition performance for neutral faces (see also Figure S2). This is consistent with the findings of previous studies that showed females have an advantage over males in face memory (Rehnman and Herlitz, 2007). In contrast, the sex of participants did not predict recognition performance for both angry and happy faces. The reason the sex of participants predicted only the recognition performance for neutral faces is unclear, and more research is needed to shed light on this issue.

One important caveat about the current findings is that ambient darkness as operationally defined here (darkness in the laboratory) likely differs in some meaningful ways from ambient darkness in other contexts. First, in many contexts, ambient darkness may serve to make expressions more difficult to see, and therefore more ambiguous, thereby naturalistically confounding the anxiety-producing effects of darkness with visual ambiguity of others' expressions. However, given that in the current work we present expressions on independently lit computer screens, the ambient darkness does not degrade the visual quality of the stimuli. If anything, lit screens in dark rooms may make the expressive stimuli more salient and distinct than they may be in lighted conditions. However, such darkness-driven increases in stimulus salience cannot easily explain the observed interactive effects of anxiety and darkness on recognition. Thus, although the current paradigm does differ in meaningful ways from many ecological contexts, these differences do not undermine the internal validity of the current work.

In summary, our study has advanced face recognition research by showing that an interactive effect of darkness and anxiety on the memory of threatening faces. Although research on the influence of contextual information on the processing of facial expressions has been neglected, the results of the present study suggest that darkness, as an ecologically valid environmental context, have significant influence on the processing of intimidating facial signal from other individuals. To promote better understanding of the mechanisms of the processing of facial expression in real life, researchers must investigate such processing under ecologically valid contexts.

## Author contributions

Satoshi F. Nakashima and Yuko Morimoto developed the study concept and design. Testing, data collections were performed by Satoshi F. Nakashima. Satoshi F. Nakashima performed the data analysis and interpretation under the supervision of Sakiko Yoshikawa. Satoshi F. Nakashima drafted the manuscript, and Yuko Morimoto, Yuji Takano, Kurt Hugenberg, and Sakiko Yoshikawa provided critical revisions. All authors approved the final version of the manuscript for submission.

### Conflict of interest statement

The authors declare that the research was conducted in the absence of any commercial or financial relationships that could be construed as a potential conflict of interest.

## References

[B1] Ackerman J. M. Becker V. D. Mortensen C. R. Sasaki T. Neuberg S. L. Kenrick D. T. (2009). A pox on the mind: disjunction of attention and memory in the processing of physical disfigurement. J. Exp. Soc. Psychol. 45, 478–485 10.1016/j.jesp.2008.12.008 19578547 PMC2699287

[B2] Adams R. B. Jr. Ambady N. Macrae C. N. Kleck R. E. (2006). Emotional expressions forecast approach-avoidance behavior. Motiv. Emot. 30, 177–186 10.1007/s11031-006-9020-2

[B3] Aiken L. S. West S. G. (1991). Multiple regression: Testing and interpreting interactions. Newbury Park; London: Sage

[B4] Anderson A. K. Yamaguchi Y. Grabski W. Lacka D. (2006). Emotional memories are not all created equal: evidence for selective memory enhancement. Learn. Mem. 13, 711–718 10.1101/lm.388906 17101871 PMC1783624

[B5] Attwood A. S. Penton-Voak I. S. Burton A. M. Munafò M. R. (2013). Acute anxiety impairs accuracy in identifying photographed faces. Psychol. Sci. 24, 1591–1594 10.1177/0956797612474021 23780726

[B6] Aviezer H. Hassin R. R. Ryan J. Grady C. Susskind J. Anderson A. (2008). Angry, disgusted, or afraid? Studies on the malleability of emotion perception. Psychol. Sci. 19, 724–732 10.1111/j.1467-9280.2008.02148.x 18727789

[B7] Bekker M. H. van Mens-Verhulst J. (2007). Anxiety disorders: sex differences in prevalence, degree, and background, but gender-neutral treatment. Gend. Med. 4, S178–S193 10.1016/S1550-8579(07)80057-X 18156102

[B8] Bradley B. P. Mogg K. Falla S. J. Hamilton L. R. (1998). Attentional bias for threatening facial expressions in anxiety: manipulation of stimulus duration. Cogn. Emot. 12, 737–753 10.1080/02699939837941110372472

[B9] Bradley B. P. Mogg K. White J. Groom C. Bono J. (1999). Attentional bias for emotional faces in generalized anxiety disorder. Br. J. Clin. Psychol. 38, 267–278 10.1348/014466599162845 10532148

[B10] Carroll J. M. Russell J. A. (1996). Do facial expressions signal specific emotions? Judging emotion from the face in context. J. Pers. Soc. Psychol. 70, 205–218 10.1037/0022-3514.70.2.205 8636880

[B11] Christianson S. Å. (1992). Emotional stress and eyewitness memory: a critical review. Psychol. Bull. 112, 284–309 10.1037/0033-2909.112.2.284 1454896

[B12] Cohen J. Cohen P. (1983). Applied Multiple Regression/Correlation Analysis for the Behavioral Sciences, 2nd Edn. Hillsdale, NJ: Lawrence Erlbaum

[B13] D'Argembeau A. Van der Linden M. (2007). Facial expressions of emotion influence memory for facial identity in an automatic way. Emotion 7, 507–515 10.1037/1528-3542.7.3.507 17683207

[B14] D'Argembeau A. Van der Linden M. Comblain C. Etienne A. M. (2003). The effects of happy and angry expressions on identity and expression memory for unfamiliar faces. Cogn. Emot. 17, 609–622 10.1080/02699930302303 29715730

[B15] Ekman P. (2003). Emotions Revealed. New York, NY: Times Books

[B16] Fox E. Russo R. Bowles R. Dutton K. (2001). Do threatening stimuli draw or hold visual attention in subclinical anxiety? J. Exp. Psychol. Gen. 130, 681–700 10.1037/0096-3445.130.4.681 11757875 PMC1924776

[B17] Fridlund A. J. (1994). Human Facial Expression: An Evolutionary View. San Diego, CA: Academic Press

[B18] Frijda N. H. (1995). Expression, emotion, neither, or both? Cogn. Emot. 9, 617–635 10.1080/02699939508408986

[B19] Grillon C. Merikangas K. R. Dierker L. Snidman N. Arriaga R. I. Kagan J. (1999). Startle potentiation by threat of aversive stimuli and darkness in adolescents: a multi-site study. Int. J. Psychophysiol. 32, 63–73 10.1016/S0167-8760(99)00002-1 10192009

[B20] Grillon C. Pellowski M. Merikangas K. R. Davis M. (1997). Darkness facilitates the acoustic startle reflex in humans. Biol. Psychiatry 42, 453–460 10.1016/S0006-3223(96)00466-0 9285081

[B21] Hansen C. H. Hansen R. D. (1988). Finding the face in the crowd: an anger superiority effect. J. Pers. Soc. Psychol. 54, 917–924 10.1037/0022-3514.54.6.917 3397866

[B22] Hietanen J. K. Leppänen J. M. (2008). Judgment of other people's facial expressions of emotions is influenced by their concurrent affective hand movements. Scand. J. Psychol. 49, 221–230 10.1111/j.1467-9450.2008.00644.x 18384495

[B23] Horley K. Williams L. M. Gonsalvez C. Gordon E. (2003). Social phobics do not see eye to eye: A visual scanpath study of emotional expression processing. J. Anxiety Disord. 17, 33–44 10.1016/S0887-6185(02)00180-9 12464287

[B24] Hugenberg K. (2005). Social categorization and the perception of facial affect: target race moderates the response latency advantage for happy faces. Emotion 5, 267–276 10.1037/1528-3542.5.3.267 16187863

[B25] Ishai A. Yago E. (2006). Recognition memory of newly learned faces. Brain Res. Bull. 71, 167–173 10.1016/j.brainresbull.2006.08.017 17113943

[B26] Johansson M. Mecklinger A. Treese A. C. (2004). Recognition memory for emotional and neutral faces: an event-related potential study. J. Cogn. Neurosci. 16, 1840–1853 10.1162/0898929042947883 15701233

[B27] Leppänen J. M. Hietanen J. K. (2003). Affect and face perception: odors modulate the recognition advantage of happy faces. Emotion 3, 315–326 10.1037/1528-3542.3.4.315 14674826

[B28] Marsh A. A. Ambady N. Kleck R. E. (2005). The effects of fear and anger facial expressions on approach-and avoidance-related behaviors. Emotion 5, 119–124 10.1037/1528-3542.5.1.119 15755225

[B29] McKelvie S. J. (1976). The role of eyes and mouth in the memory of a face. Am. J. Psychol. 89, 311–323 10.2307/1421414

[B30] Meeren H. K. van Heijnsbergen C. C. de Gelder B. (2005). Rapid perceptual integration of facial expression and emotional body language. Proc. Natl. Acad. Sci. U.S.A. 102, 16518–16523 10.1073/pnas.0507650102 16260734 PMC1283446

[B31] Nakashima S. F. Langton S. R. Yoshikawa S. (2012). The effect of facial expression and gaze direction on memory for unfamiliar faces. Cogn. Emot. 26, 1316–1325 10.1080/02699931.2011.619734 22077759

[B32] Ogawa T. Oda M. Yoshikawa S. Akamatsu S. (1997). Evaluation of facial expressions differing in face angles -constructing a database of facial expressions- (In Japanese with English abstract), in Technical Report of Institute of Electronics, Information and Communication Engineers (IEICE), HIP, 47–52

[B33] Rehnman J. Herlitz A. (2007). Women remember more faces than men do. Acta Psychol. (Amst.) 124, 344–355 10.1016/j.actpsy.2006.04.004 16764811

[B34] Righart R. de Gelder B. (2008). Recognition of facial expressions is influenced by emotional scene gist. Cogn. Affect. Behav. Neurosci. 8, 264–272 10.3758/CABN.8.3.264 18814463

[B35] Rohner J. C. (2002). The time-course of visual threat processing: high trait anxious individuals eventually avert their gaze from angry faces. Cogn. Emot. 16, 837–844 10.1080/02699930143000572

[B36] Schaller M. Park J. H. Mueller A. (2003). Fear of the dark: Interactive effects of beliefs about danger and ambient darkness on ethnic stereotypes. Pers. Soc. Psychol. Bull. 29, 637–649 10.1177/0146167203029005008 15272996

[B37] Sergerie K. Lepage M. Armony J. L. (2005). A face to remember: emotional expression modulates prefrontal activity during memory formation. Neuroimage 24, 580–585 10.1016/j.neuroimage.2004.08.051 15627601

[B38] Shimizu H. Imae K. (1981). State-trait anxiety inventory no nihongoban (daigakuseiyo) no sakusei [Making of the Japanese college student version of the state-trait anxiety inventory]. Kyoiku Shinrigaku Kenkyu 29, 348–353

[B39] Spielberger C. D. Gorsuch R. L. Lushene R. E. (1970). Manual for the State-Trait Anxiety Inventory (Self-Evaluation Questionnaire). Palo Alto, CA: Consulting Psychology Press

[B40] Springer U. S. Rosas A. McGetrick J. Bowers D. (2007). Differences in startle reactivity during the perception of angry and fearful faces. Emotion 7, 516–525 10.1037/1528-3542.7.3.516 17683208

[B41] Takahashi T. Ikeda K. Ishikawa M. Tsukasaki T. Nakama D. Tanida S. (2004). Social stress-induced cortisol elevation acutely impairs social memory in humans. Neurosci. Lett. 363, 125–130 10.1016/j.neulet.2004.03.062 15172099

[B42] Thayer J. Johnsen B. H. (2000). Sex differences in judgement of facial affect: a multivariate analysis of recognition errors. Scand. J. Psychol. 41, 243–246 10.1111/1467-9450.00193 11041306

[B43] Van den Stock J. de Gelder B. (2012). Emotional information in body and background hampers recognition memory for faces. Neurobiol. Learn. Mem. 97, 321–325 10.1016/j.nlm.2012.01.007 22406473

[B44] Van den Stock J. Righart R. de Gelder B. (2007). Body expressions influence recognition of emotions in the face and voice. Emotion 7, 487–494 10.1037/1528-3542.7.3.487 17683205

[B45] Wickens T. D. (2002). Elementary Signal Detection Theory. Oxford: Oxford University Press

