# Supplementary figures and images for: Faces in the dark: interactive effects of darkness and anxiety on the memory for threatening faces
Source: Front Psychol. 2014 Oct 2;5:1091. doi: 10.3389/fpsyg.2014.01091 (PMC4183089; doi:10.3389/fpsyg.2014.01091)

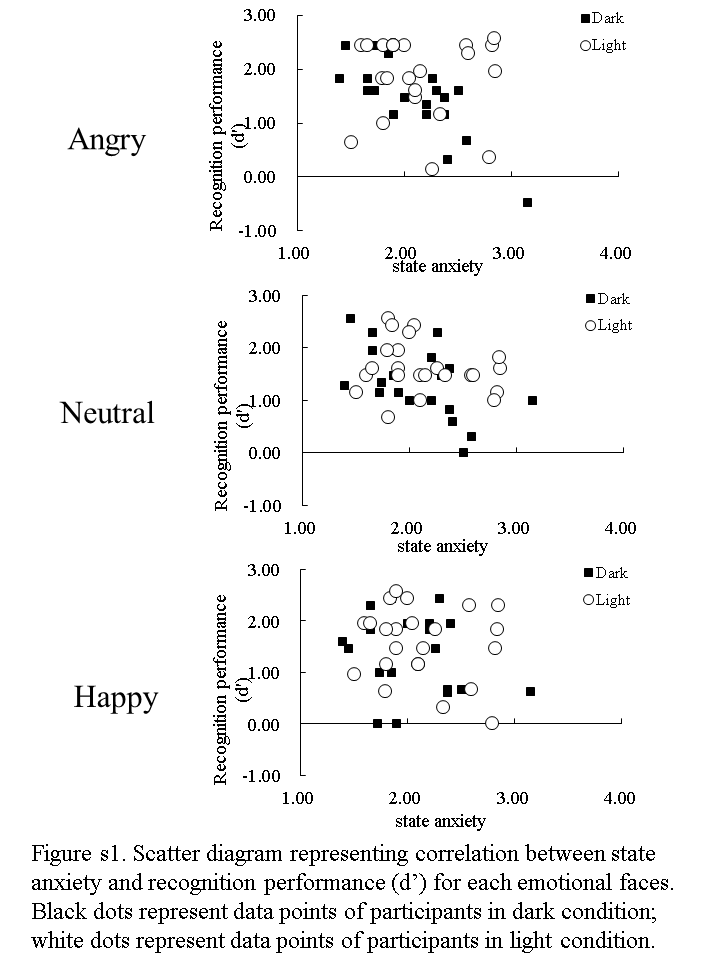

Supplement: Supplementary file 1 [file Image1.PNG]

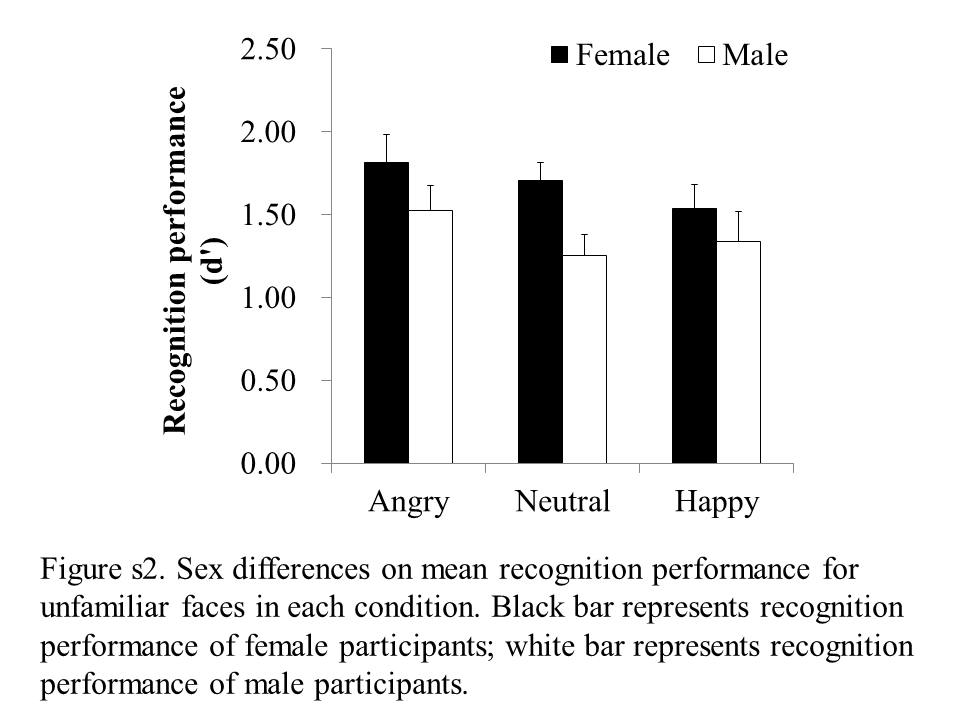

Supplement: Supplementary file 2 [file Image2.PNG]
